# Основные эпидемиологические показатели сахарного диабета 1 типа у детей в Российской Федерации за 2014–2023 годы

**DOI:** 10.14341/probl13515

**Published:** 2024-11-04

**Authors:** Д. Н. Лаптев, О. В. Безлепкина, Е. Л. Шешко, Г. А. Александрова, О. В. Чумакова, Н. М. Крестовская, А. Ш. Кулаев, В. А. Петеркова

**Affiliations:** Национальный медицинский исследовательский центр эндокринологии; Национальный медицинский исследовательский центр эндокринологии; Департамент медицинской помощи детям, службы родовспоможения и общественного здоровья Минздрава России; Департамент мониторинга, анализа и стратегического развития здравоохранения Минздрава России; Департамент медицинской помощи детям, службы родовспоможения и общественного здоровья Минздрава России; Департамент медицинской помощи детям, службы родовспоможения и общественного здоровья Минздрава России; Департамент медицинской помощи детям, службы родовспоможения и общественного здоровья Минздрава России; Национальный медицинский исследовательский центр эндокринологии

**Keywords:** сахарный диабет 1 типа, дети, заболеваемость, распространенность

## Abstract

**ОБОСНОВАНИЕ:**

ОБОСНОВАНИЕ. Сахарный диабет 1 типа (СД1) является наиболее распространенной формой сахарного диабета в детском возрасте, где, в отличие от взрослых, на его долю приходится более 90% всех случаев диабета. Постоянное изменение эпидемиологии СД1 с существенными отличиями в различных популяциях и регионах требует систематического сбора и анализа данных для своевременного мониторинга трендов СД1 у детей.

**ЦЕЛЬ:**

ЦЕЛЬ. Анализ основных эпидемиологических показателей СД1 у детей в Российской Федерации за последние 10 лет — с 2014 по 2023 гг.

**МАТЕРИАЛЫ И МЕТОДЫ:**

МАТЕРИАЛЫ И МЕТОДЫ. Объектом исследования являлись данные, полученные из формы федерального статистического наблюдения №12 «Сведения о числе заболеваний, зарегистрированных у пациентов, проживающих в районе обслуживания медицинской организации», за период с 2014 по 2023 гг.

Были проанализированы показатели распространенности (общее количество зарегистрированных заболеваний) и заболеваемости (случаи с впервые в жизни установленным диагнозом) СД1 (код по МКБ-10: Е10) у детей в трех возрастных группах: от 0 до 14 лет, от 15 до 17 лет и объединенно от 0 до 17 лет (включительно).

**РЕЗУЛЬТАТЫ:**

РЕЗУЛЬТАТЫ. За анализируемый период распространенность СД1 равномерно увеличивалась с 238,6 в 2014 г. до 374,2 случая на 100 000 детского населения в 2023 г. Распространенность СД1 в подростковом возрасте от 15 до 17 лет закономерно была выше, чем у детей, и составила 120,3–203,2 случая на 100 000 подростков, в то время как у детей до 14 лет распространенность составила 100,1–172,2 случая на 100 000 детей. Ежегодный прирост распространенности СД1 в среднем составил 6,3% (95% ДИ 4,9–7,8). Заболеваемость СД1 за анализируемый период составила 19,1–27,2 случая на 100 000 детского населения и также имела общую тенденцию к ежегодному приросту новых случаев. Вместе с тем за последние три года отмечается относительная стабилизация показателей заболеваемости на уровне 26,5–27,2 на 100 000 детского населения. Ежегодный прирост заболеваемости в среднем составил 4,9% (95% ДИ 0,9–8,9). Наибольший прирост заболеваемости СД1 отмечается в регионах с низкой заболеваемостью.

**ЗАКЛЮЧЕНИЕ:**

ЗАКЛЮЧЕНИЕ. Эпидемиология СД1 в Российской Федерации характеризуется значительными региональными и динамическими изменениями. За период 2014–2023 гг. заболеваемость СД1 у детей существенно выросла, увеличиваясь ежегодно в среднем на 5%, при этом наблюдается относительное замедление и стабилизация показателей заболеваемости за последние три года.

## Abstract (en)

**BACKGROUND:**

BACKGROUND: Type 1 diabetes mellitus (T1DM) is the most common form of diabetes mellitus in childhood, where, unlike in adults, it accounts for more than 90% of all cases of diabetes. The constant change in the epidemiology of T1DM with significant differences in populations and regions requires systematic data collection and analysis for timely monitoring of T1DM trends.

**AIM:**

AIM: Analysis of the main epidemiological indicators of T1DM in children in the Russian Federation over the past 10 years — from 2014 to 2023.

**MATERIALS AND METHODS:**

MATERIALS AND METHODS: The object of the study was the data obtained from the federal statistical observation form No. 12 «Information on the number of diseases registered in patients living in the service area of a medical organization» for the period from 2014 to 2023. The prevalence (total number of registered cases) and incidence (cases with a diagnosis established for the first time) of T1DM (ICD-10 code: E10) were analyzed in children in three age groups: from 0 to 14 years, from 15 to 17 years, and combined from 0 to 17 years (inclusive).

**RESULTS:**

RESULTS: Over the analyzed period, the prevalence of T1DM increased steadily from 238.6 in 2014 to 374.2 cases per 100,000 children in 2023. The prevalence of T1DM in adolescents from 15 to 17 years was higher than in children and amounted to 120.3-203.2 cases per 100,000 adolescents, while in children under 14 years of age, the prevalence was 100.1–172.2 cases per 100,000 children. The annual increase in the prevalence of T1DM averaged 6.3% (95% CI 4.9–7.8). The incidence of T1DM during the analyzed period was 19.1–27.2 cases per 100,000 children and also had a general tendency toward an annual increase in new cases. At the same time, over the past three years, there has been a relative stabilization of incidence rates at 26.5–27.2 per 100,000 children. The annual increase in incidence averaged 4.9% (95% CI 0.9–8.9). The greatest increase in the incidence of T1DM was observed in regions with low incidence.

**CONCLUSION:**

CONCLUSION: The epidemiology of T1DM in the Russian Federation is characterized by significant regional and dynamic changes. Over the period 2014-2023, the incidence of type 1 diabetes in children has increased significantly, increasing annually by an average of 5%, while there has been a relative stabilization of incidence rates over the past three years.

## ОБОСНОВАНИЕ

Сахарный диабет 1 типа (СД1) — одна из наиболее распространенных эндокринопатий в детском возрасте, что в сочетании с высокой социальной значимостью делает заболевание одной из наиболее актуальных проблем детской эндокринологии. СД1 является наиболее распространенной формой сахарного диабета в детском возрасте, где, в отличие от взрослых, на его долю приходится более 90% всех случаев диабета.

По данным IDF, на сегодняшний день в мире насчитывается порядка 8,75 млн людей с СД1, из которых 1,5 млн — в возрасте до 20 лет, а число новых случаев в этой возрастной группе ежегодно составляет порядка 200 тысяч [1][2]. При этом заболеваемость СД1 значительно варьирует в зависимости от региона и континента. Наибольшая заболеваемость традиционно характерна для скандинавских стран, в частности в Финляндии у детей регистрируется более 50 новых случаев СД1 на 100 000 [3]. В то же время в азиатских странах отмечается наименьшая частота СД1, и заболеваемость обычно не превышает 2–3 случая на 100 000 детского населения [4]. Помимо региональных особенностей, заболеваемость СД1 варьирует в зависимости от возраста, пола и времени года [5–8]. Кроме того, во многих странах за последние годы отмечается прирост новых случаев СД1, и ежегодный рост заболеваемости составляет порядка 3–4% [9].

Постоянное изменение эпидемиологии СД1 с существенными отличиями в различных популяциях и регионах требует систематического сбора и анализа данных для своевременного мониторинга трендов СД1 у детей. В Российской Федерации ведется Федеральный регистр сахарного диабета, вместе с тем опубликованные данные по СД1 у детей не охватывают достаточно длительные периоды времени, чтобы оценить долгосрочные тренды [10][11].

## ЦЕЛЬ ИССЛЕДОВАНИЯ

Целью данной работы был анализ основных эпидемиологических показателей СД1 у детей в Российской Федерации за последние 10 лет — с 2014 по 2023 гг.

## МАТЕРИАЛЫ И МЕТОДЫ

Дизайн исследования

Одноцентровое, одномоментное, ретроспективное исследование. Объектом исследования были данные, полученные из формы федерального статистического наблюдения №12 «Сведения о числе заболеваний, зарегистрированных у пациентов, проживающих в районе обслуживания медицинской организации» за период с 2014 по 2023 гг.

Методы

Были проанализированы показатели распространенности (общее количество зарегистрированных заболеваний) и заболеваемости (случаи с впервые в жизни установленным диагнозом) СД1 (код по МКБ-10: Е10) у детей в трех возрастных группах: от 0 до 14 лет, от 15 до 17 лет и объединенно от 0 до 17 лет (включительно).

В исследование были включены данные по субъектам Российской Федерации, для которых имелась информация по заболеваемости и распространенности за период с 2014 по 2023 гг.

Данные об общей численности населения по возрасту на начало соответствующего года были получены из бюллетеней Федеральной службы государственной статистики (Росстат, rosstat.gov.ru).

Статистический анализ

Показатели распространенности и заболеваемости были рассчитаны для каждого календарного года по отношению к общей популяции детей аналогичного возраста и представлены в виде числа случаев СД1 на 100 000 детского населения. 95% доверительные интервалы (ДИ) для показателей распространенности и заболеваемости были рассчитаны исходя из предположения, что они имеют распределение Пуассона. Данные по приросту заболеваемости и распространенности представлены в виде средних значений и 95% ДИ.

Этическая экспертиза

Протокол исследования одобрен локальным комитетом по этике ФГБУ «НМИЦ эндокринологии» Минздрава России (выписка из протокола №11 от 13.06.2024).

## РЕЗУЛЬТАТЫ

Распространенность

За анализируемый период распространенность СД1 равномерно увеличивалась с 238,6 в 2014 г. до 374,2 случая на 100 000 детского населения в 2023 г. (рис. 1). Распространенность СД1 в подростковом возрасте от 15 до 17 лет закономерно была выше, чем у детей, и составила 120,3–203,2 случая на 100 000 подростков, в то время как у детей до 14 лет распространенность составила 100,1–172,2 случая на 100 000 детей.

В среднем распространенность СД1 за период 2014–2023 гг. составила 157,9 (95% ДИ 156,5–159,4) на 100 000 детского населения, увеличившись с 135,6 (134,3–137,0) в период 2014–2018 гг. до 179,2 (95% ДИ 177,7–180,7) на 100 000 детского населения в 2019–2023 гг. (табл 1). Ежегодный прирост распространенности в среднем составил 6,3% (95% ДИ 4,9–7,8), при этом в целом средний прирост распространенности не различался между детьми и подростками и в различные временные периоды.

Заболеваемость

Заболеваемость СД1 за анализируемый период составила 19,1–27,2 случая на 100 000 детского населения и также имела общую тенденцию к ежегодному приросту новых случаев (рис. 2). В то же время в период с 2016 по 2018 гг. у подростков и в меньшей степени у детей наблюдалось снижение заболеваемости. Другим заметным трендом стала стабилизация заболеваемости СД1 в целом у детей 0–17 лет в последние годы — в период с 2021 по 2023 гг., при этом у детей 0–14 и подростков 15–17 лет отмечалась несколько разнонаправленная динамика.

Средняя заболеваемость СД1 за весь анализируемый период составила 23,1 (95% ДИ 22,5–23,6) на 100 000 детского населения, увеличившись с 20,4 (95% ДИ 19,9–20,9) в период 2014–2018 до 25,6 (95% ДИ 25,0–26,2) на 100 000 детского населения в 2019–2023 (табл 1, 2). Ежегодный прирост заболеваемости в среднем составил 4,9% (95% ДИ 0,9–8,9), существенно не изменившись за анализируемые периоды времени. Аналогично ежегодный прирост заболеваемости не различался между детьми и подростками, в том числе в разрезе анализируемых периодов времени.

Заболеваемость по субъектам Российской Федерации

Заболеваемость СД1 значительно различалась между субъектами Российской Федерации (рис. 3). Наибольшая заболеваемость за анализируемый период отмечалась в Ненецком автономном округе, Санкт-Петербурге и Ленинградской области — 63,4, 39,8 и 38 на 100 000 детского населения соответственно. Наименьшая заболеваемость зафиксирована в Республике Тыва, Чеченской Республике и Республике Калмыкия — 5, 7,1 и 8,1 на 100 000 детского населения соответственно. В целом можно отметить тенденцию к большей заболеваемости СД1 в северных и северо-западных регионах страны, и к меньшей заболеваемости СД1 в Северо-Кавказском регионе.

Средний прирост заболеваемости за период 2014–2023 еще более существенно варьировал между различными регионами (рис. 4). Наибольший прирост заболеваемости отмечался в Чеченской Республике, Республике Алтай и Калининградской области: +48% (95% ДИ -30,7–126,6), +35,6% (95% ДИ -30,6–101,9) и +34,5% (95% ДИ -28,8–97,8) соответственно. Наименьший прирост заболеваемости зарегистрирован в Ненецком автономном округе, Ярославской и Нижегородской областях: -4,2% (95% ДИ -12,6–4,1), +1,8% (95% ДИ -8,2–11,7) и +2,3% (95% ДИ -11,5–16,1) соответственно.

**Figure:**
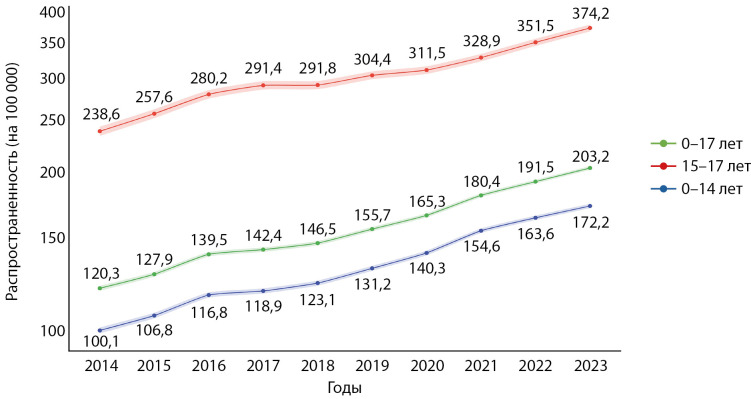
Рисунок 1. Динамика показателей распространенности СД1 у детей за период 2014–2023 гг. Данные представлены в виде числа случаев на 100 000 населения соответствующего возраста и 95% ДИ.

**Table:** Таблица 1. Сравнительная характеристика средних показателей распространенности и заболеваемости, а также относительного прироста за различные периоды. Данные представлены в виде числа случаев на 100 000 населения соответствующего возраста и 95% ДИ или % и 95% ДИ (для показателей прироста)

Распространенность
Возраст	2014–2018	2019–2023	2014–2023
Общая	Прирост	Общая	Прирост	Общая	Прирост
0–14лет	113,4(112,1–114,8)	6,3(3,5–9,1)	152,3(150,8–153,8)	7,0(5,5–8,5)	133,3(131,8–134,7)	6,6(5,0–8,2)
15–17лет	272,1(267,0–277,2)	6,1(3,0–9,3)	334,7(329,4–340,1)	5,1(3,7–6,6)	305,0(299,8–310,3)	5,6(3,9–7,4)
0–17лет	135,6(134,3–137,0)	5,9(3,3–8,5)	179,2(177,7–180,7)	6,8(5,7–7,8)	157,9(156,5–159,4)	6,3(4,9–7,8)
Заболеваемость
Возраст	2014–2018	2019–2023	2014–2023
Общая	Прирост	Общая	Прирост	Общая	Прирост
0–14лет	19,5(18,9–20,0)	4,2(-1,6–10,0)	24,9(24,3–25,5)	5,4(-0,7–11,5)	22,2(21,6–22,8)	4,8(0,6–9,0)
15–17лет	25,8(24,3–27,4)	6,2(-3,0–15,4)	29,9(28,3–31,5)	4,9(2,9–6,8)	28,0(26,4–29,6)	5,5(0,8–10,3)
0–17лет	20,4(19,9–20,9)	4,4(-1,4–10,2)	25,6(25,0–26,2)	5,4(0,0–10,7)	23,1(22,5–23,6)	4,9(0,9–8,9)

**Table:** Таблица 2. Динамика показателей (абсолютные значения) распространенности и заболеваемости по годам и возрастным группам

	Распространенность абс.	Заболеваемость абс.
0–14 лет	15–17 лет	0–17 лет	0–14 лет	15–17 лет	0–17 лет
2014	23 409	9520	32 929	4361	866	5227
2015	26 054	10 214	36 268	4578	1018	5596
2016	29 185	11 292	40 477	5118	1161	6279
2017	30 374	11 729	42 103	5004	1116	6120
2018	31 769	12 147	43 916	5125	1054	6179
2019	34 042	13 008	47 050	5912	1151	7063
2020	36 399	13 810	50 209	6052	1260	7312
2021	40 023	14 786	54 809	6790	1376	8166
2022	42 236	15 850	58 086	6822	1418	8240
2023	43 985	17 333	61 318	6524	1485	8009

**Figure:**
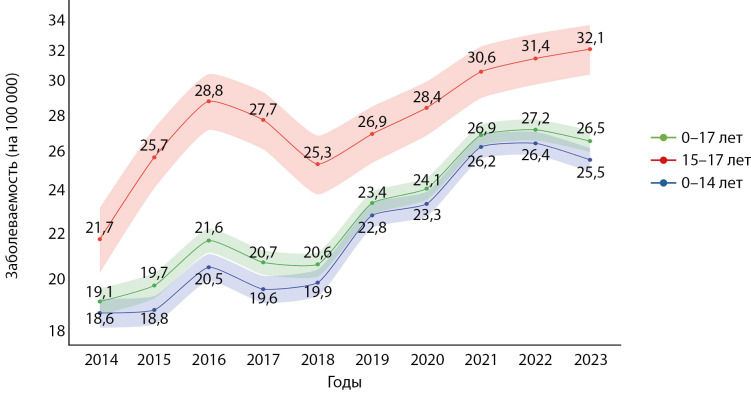
Рисунок 2. Динамика показателей заболеваемости СД1 у детей за период 2014–2023 гг. Данные представлены в виде числа случаев на 100 000 населения соответствующего возраста и 95% ДИ.

**Figure:**
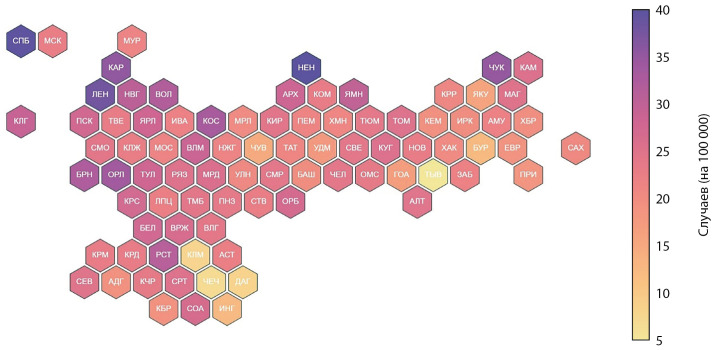
Рисунок 3. Средние показатели заболеваемости СД1 по субъектам за период 2014–2023 гг. Данные представлены в виде числа случаев на 100 000 детского населения в возрасте до 18 лет.

**Figure:**
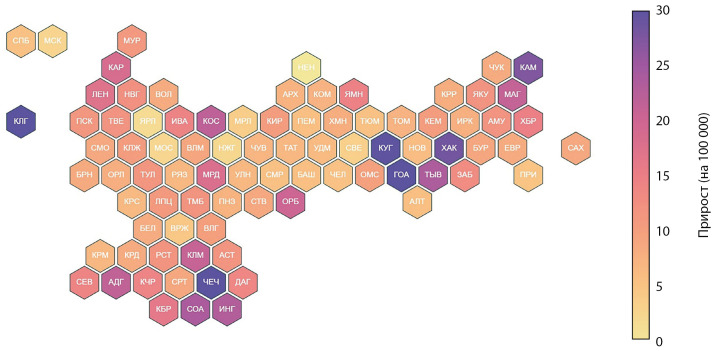
Рисунок 4. Средний ежегодный прирост заболеваемости СД1 по субъектам за период 2014–2023. Данные представлены в виде числа случаев на 100 000 детского населения в возрасте до 18 лет.

## Обсуждение

СД1 — наиболее распространенная форма сахарного диабета в детском возрасте, что определяет высокую актуальность изучения и оценки эпидемиологических характеристик. Социальная значимость СД1 обусловлена повышенным риском заболеваемости и смертности от осложнений и как следствие инвалидизацией и значительным снижением продолжительности жизни пациентов, а также социальными и психологическими проблемами, с которыми сталкиваются семьи детей с СД1.

В данном исследовании проведена оценка динамики заболеваемости и распространенности СД1 за 10-летний период у детей и подростков в Российской Федерации. Полученные данные свидетельствуют о тенденции к ежегодному увеличению заболеваемости во всех возрастных группах в среднем на уровне 5% за анализируемый период и, как следствие, роста общего числа случаев СД1. За последние десятилетия отмечался глобальный рост заболеваемости СД1 [1][12], в то же время в последние годы во многих популяциях наблюдается замедление этой тенденции и выход на плато по показателям заболеваемости [7][13]. Точные причины роста заболеваемости не вполне понятны, но так как возникновение даже минимальных изменений в генетике потребует смены многих поколений, то наиболее вероятной причиной является влияние средовых факторов: изменение микробиома, вирома, увеличение случаев ожирения, стрессовые и другие индустриальные воздействия и пр. [14].

Аналогично другим исследованиям, по нашим данным за последние несколько лет (2021–2023 гг.), заболеваемость СД1 у детей и в меньшей степени у подростков находится на относительно стабильном уровне: 25,5–26,2 на 100 000 у детей, 30,6–32,1 на 100 000 у подростков и 26,5–27,2 на 100 000 в общей группе. Стабилизация заболеваемости, с одной стороны, может указывать на цикличность в периодах роста и снижение заболеваемости [7][15], с другой стороны — на стабилизацию воздействия триггерных факторов, связанных с изменившейся внешней средой.

Заболеваемость СД1 имеет сложный характер и значительно варьирует не только в разных странах, но и в различных регионах в пределах одной страны, что подчеркивает важность этнических и средовых факторов в возникновении и прогрессировании аутоиммунного процесса. Как и в предыдущие годы [10], так и по данным этой работы, наибольшая заболеваемость СД1 у детей в Российской Федерации наблюдается в северо-западных регионах (город Санкт-Петербург, Ленинградская область, Ненецкий автономный округ), а наименьшая — в северокавказских (Республика Чечня, Республика Дагестан, Республика Ингушетия, Республика Калмыкия) и восточносибирских (республика Тыва) регионах. В то же время, несмотря на сравнительно более низкую общую заболеваемость в этих субъектах, для многих из них характерен наиболее выраженный прирост заболеваемости, в том числе по сравнению с северо-западными регионами. В частности, в Чеченской Республике, Республике Ингушетия, Республике Калмыкия прирост заболеваемости составил в среднем +48% (-30,7–126,6), +23,1 (-4,6–50,8) и +20,9 (-22,1–63,8) соответственно, при среднем приросте заболеваемости по Российской Федерации 4,9% (0,9–8,9).

Существенные различия в заболеваемости в различных регионах, очевидно, обусловлены многими обстоятельствами, включающими особенности популяционной генетики регионов [16–18], характера и интенсивности воздействия средовых факторов [19]. Например, на территориях Российской Федерации имеются значительные различия и особенности в циркулирующих видах энтеровируса [20], который рассматривается как один из основных кандидатов, инициирующих аутоиммунную реакцию [21]. Значительный прирост заболеваемости в регионах с исходно низкими показателями могут дополнительно подчеркивать важную роль средовых факторов в формировании и прогрессировании аутоиммунного процесса. Например, последние годы характеризуются заметными изменениями в структуре и частоте инфекционных заболеваний [22], также, очевидно, значительный вклад внесла пандемия COVID 19 [23], сохраняется общая мобильность населения.

Учитывая высокую социальную значимость СД1 у детей, а также ресурсы, затрачиваемые системой здравоохранения на лечение, необходима регулярная оценка и анализ эпидемиологических характеристик этого заболевания. Учитывая динамически меняющуюся заболеваемость СД1, только длительное наблюдение позволит установить закономерные тенденции и динамику.

## ЗАКЛЮЧЕНИЕ

Эпидемиология СД1 в Российской Федерации характеризуется значительными региональными и динамическими изменениями. За период 2014–2023 гг. заболеваемость СД1 у детей существенно выросла, увеличиваясь ежегодно в среднем на 5%, при этом наблюдается относительное замедление и стабилизация показателей заболеваемости за последние три года. Рост заболеваемости отразился на общей распространенности СД1, которая выросла примерно в 1,5 раза. Сохраняется значительная неоднородность в заболеваемости СД1 по регионам, при этом имеется тенденция к более значительному приросту показателя в регионах с низким уровнем заболеваемости. Только более длительное наблюдение и анализ позволят оценить данные тренды.

## Дополнительная информация

Источники финансирования. Исследование выполнено при поддержке Министерства науки и высшего образования Российской Федерации (соглашение №075-15-2024-645).

Конфликт интересов. Авторы декларируют отсутствие явных и потенциальных конфликтов интересов, связанных с содержанием настоящей статьи.

Участие авторов. Все авторы одобрили финальную версию статьи перед публикацией, выразили согласие нести ответственность за все аспекты работы, подразумевающую надлежащее изучение и решение вопросов, связанных с точностью или добросовестностью любой части работы.
